# Specific Patterns in Correlations of Super-Short Tandem Repeats (SSTRs) with G+C Content, Genic and Intergenic Regions, and Retrotransposons on All Human Chromosomes

**DOI:** 10.3390/genes15010033

**Published:** 2023-12-25

**Authors:** Lukas Henn, Aaron Sievers, Michael Hausmann, Georg Hildenbrand

**Affiliations:** 1 Kirchhoff Institute for Physics, Heidelberg University, INF 227, 69117 Heidelberg, Germany; l.henn@stud.uni-heidelberg.de (L.H.); aaron.sievers@med.uni-heidelberg.de (A.S.); hausmann@kip.uni-heidelberg.de (M.H.); 2 Institute for Human Genetics, University Hospital Heidelberg, INF 366, 69117 Heidelberg, Germany; 3 Faculty of Engineering, University of Applied Science Aschaffenburg, Würzburger Str. 45, 63743 Aschaffenburg, Germany

**Keywords:** *k-mer*, tandem repeats, transposons

## Abstract

The specific characteristics of *k-mer* words (2 ≤ k ≤ 11) regarding genomic distribution and evolutionary conservation were recently found. Among them are, in high abundance, words with a tandem repeat structure (repeat unit length of 1 bp to 3 bp). Furthermore, there seems to be a class of extremely short tandem repeats (≤12 bp), so far overlooked, that are non-random-distributed and, therefore, may play a crucial role in the functioning of the genome. In the following article, the positional distributions of these motifs we call super-short tandem repeats (SSTRs) were compared to other functional elements, like genes and retrotransposons. We found length- and sequence-dependent correlations between the local SSTR density and G+C content, and also between the density of SSTRs and genes, as well as correlations with retrotransposon density. In addition to many general interesting relations, we found that SINE Alu has a strong influence on the local SSTR density. Moreover, the observed connection of SSTR patterns to pseudogenes and -exons might imply a special role of SSTRs in gene expression. In summary, our findings support the idea of a special role and the functional relevance of SSTRs in the genome.

## 1. Introduction

### STRs/SSTRs and k-mer Analysis

While the DNA sequence analysis of the human genome initially focused on the protein-coding fractions of the genome, recently, the search for patterns and their potential functions, including intronic and intergenic sequences [1,2,3,4], as well as non-coding but functional elements (e.g., retrotransposons [5,6,7]), was intensified. A comparative analysis of the sequence pattern has become an effective approach with which to identify potentially functional elements, when a direct functional analysis is not possible (e.g., without a promising hypothesis) or not feasible (e.g., by technical or financial limitations). The general idea of a comparative sequence analysis is that features with no relevant potential for genome functioning are expected to change over time due to genetic drifts [8]. Therefore, the evolutionary conservation of sequence features might imply some form of functionality of the respective features. In most studies, conservation is defined by scores, derived by powerful alignment-based algorithms (e.g., GERP++ [9] or BLAST [10]). These algorithms were very effective in finding relatively large, conserved regions within protein-coding sequences, but cannot be used to detect relatively small, very diffuse, and repetitive features (e.g., conservation of repeat density) within non-coding regions. However, such motifs may be important determinants of the conserved functional features for which the representation (encoding) within DNA sequences is not yet completely understood (e.g., 3D chromatin organization and transcriptional regulation).

The approach we use in this study to find the conserved sequence patterns is the so-called *k-mer* analysis [11]. As the name suggests, this technique focuses on frequencies of DNA words of length k. The analyzed DNA sequences are converted into the so-called *k-mer* spectra by counting the number of appearances of each possible *k-mer* word inside the respective sequences. The *k-mer* analysis has already been used to identify interesting patterns, potentially related to biological functions like gene expression and chromatin organization [11,12,13,14,15,16]. While analyzing the *k-mer* spectra [13], it is commonplace for the most frequent and impactful *k-mer* words to show repetitive internal patterns. For example, the frequencies of *k-mer* words like “AAAAA” or “AGAGA” often dominate the results of the *k-mer* analysis. This overrepresentation of repetitive *k-mer* words in previous results motivated the more detailed analysis of repetitive *k-mer* words presented in this study. Here, we have not limited the analysis to a constant word length but analyzed DNA words with repetitive internal patterns up to 12 bp length. Considering these repetitive patterns, these DNA words are a subclass of short tandem repeats (STRs). STRs are repetitive DNA sequences characterized by a specific repeat unit, which is repeated multiple times in tandem. An STR is characterized by its repeat unit (e.g., AG) and its length (e.g., 5 bp). It can be encoded as (X)_n_, with X representing the repeat motif and n the number of the repeat motif in the STR (e.g., (AG)_5_). STRs were found to be important in fields such as cancer diagnosis or kinship analysis, but are also known to impact chromatin organization and gene expression [17,18].

In previous studies, repeat units of length 1 bp and 2 bp often dominated the *k-mer* spectra [12]. In this study, we focused on nucleotide and dinucleotide repeats. According to the length of patterns found in previous studies [12,13], the total length of the analyzed repeats was limited to sizes from 2 bp to 12 bp.

Though initially overlooked, interest in STRs has increased in recent years [18]. While STRs are more and more commonly analyzed, this specific subclass is often neglected in other studies, due to the very short total length and, therefore, very high frequencies within genomic sequences. To distinguish between the typical understanding of an STR (with varying definitions, often implying total lengths of minimum 5–100 bp), we will use the term super-short tandem repeat (SSTR) for the relatively short elements analyzed in this study. 

To overcome the difficulties resulting from the very high frequencies of SSTRs in the human genome, we limited our analysis to the comparison of local SSTR densities (resolution of 100 kbp) with densities of other elements or features. These local densities were correlated using the Pearson correlation coefficient to determine similarity [19]. In comparison to former studies, this correlation of local densities reincorporates part of the information dropped in the *k-mer* analysis approach [11]. 

The local density of SSTRs was correlated with the G+C content in the first step, since the G+C content was well-known to be associated with several functional features [20,21,22,23,24].

In the second step, the local density of SSTRs was correlated with the local densities of different functional elements. These elements include different classes of genes (protein coding, non-protein coding, and pseudogenes) and retrotransposons, which were recently found to have an impact on gene expression and chromatin organization [25].

## 2. Materials and Methods

### 2.1. Materials

#### 2.1.1. NCBI and RefSeq

Sequence data and annotations used in this study were downloaded from the NCBI website [26]. We used data from the RefSeq database, including genomic DNA sequences and annotated features important for deriving subclasses of genes. Additionally, NCBI also provides RepeatMasker files [27] including transposon annotations. Since the sequences used in this study include sequencing gaps, telomere-to-telomere sequences [28] might have been preferable for this study. We did not use telomere-to-telomere sequences because they were not (completely) available when we started this study. 

#### 2.1.2. Super-Short Tandem Repeats

All analyzed SSTRs are derived from the unmasked genome sequences found within NCBI Genbank files provided by the RefSeq Database [26]. Their positions and lengths were extracted using a software package called *Oligo* [11]. This Python- and C++-based software package allows for convenient processing of Genbank and Repeatmasker files. We used *Oligo* for searching and processing SSTRs, for the correlations of SSTR densities and other element densities, and for visualization. A complete list of used files and accession numbers can be found under Supplementary Materials (see Table S1).

All SSTRs were categorized based on their repeat unit, length, and the chromosome they are located on. As the focus of this article were nucleotide and dinucleotide repeats forming SSTRs, all 16 possible repeat units were analyzed separately. 

For deriving the frequencies of SSTRs, we only considered the longest possible SSTR; e.g., AGAGAG was only considered for a length of 6 bp, while the included shorter SSTRs (e.g., AGAG and AG) were not considered.

#### 2.1.3. Gene Categories

All gene categories were derived from NCBI Genbank File annotations from the RefSeq Database using *Oligo* [11]. Their positions and lengths were either directly determined from the corresponding annotation type or derived from combinations or subsets of these annotations.

Genic and intergenic regions were determined. Genic regions correspond to all areas covered by any gene annotation on at least one DNA strand. Intergenic regions are defined as all non-genic regions.

These regions were further divided. Genic regions were subdivided into protein-coding (pc) genes, non-protein coding (npc) genes, and pseudogenes. Pc genes are all genes that contain another annotation of either mRNA or CDS. Pseudogenes are defined as all gene annotations containing the “\pseudo” qualifier. Npc genes are all genes not covered by the two other categories, containing annotations such as ncRNA, tRNA, rRNA, and miscRNA. All mentioned subcategories were also analyzed in npc genes but the small total number of members in these categories did not allow statistically significant results. Accordingly, npc was only analyzed as one gene category.

Pc, npc, and pseudogenes were further split into exonic and intronic areas. Pc exons are all areas inside pc genes that are either annotated as CDS or mRNA. Npc exons work the same way but with ncRNA, tRNA, rRNA, and miscRNA. Pseudo exons are made up of type miscRNA and exon. However, both types also need the qualifier “\pseudo”. Introns for all three gene categories are defined as the non-exonic region in their respective genes.

#### 2.1.4. Retrotransposons

Retrotransposon position and length were derived from RepeatMasker files, originating from the RefSeq Database, using *Oligo* [11].

The columns’ repeat class/family were used to identify retrotransposon types (e.g., Alu).

### 2.2. Methods

We derived the positions and lengths for all SSTRs from genome sequences using *Oligo*. As described above, we derived positions of genes and retrotransposons. As we are interested in the similarity between local densities of these elements, two more data-processing steps were applied. First, instead of looking at exact positions, each chromosome was subdivided into segments of equal size. For SSTRs and retrotransposons, the frequencies per segment were used to determine their local density, by dividing it by the segment size. Considering the wide range of different lengths of genes, instead of the density, we used the coverage per segment. We define the coverage as the content of the segment covered by the respective element on at least one DNA strand (e.g., if half of the nucleotides in a segment were part of a gene, the coverage of genes would be 50%). If an element only partially overlaps with a segment, we only considered the overlapping parts for deriving the coverage. Similarly, the G+C content was derived from the sequences.

Using these local densities, the similarity was determined via correlation using the Pearson correlation coefficient. This process results in one value between −1 and 1 per correlation pair, with −1 for full anti-correlation and 1 for full correlation. The segment size underlying the segmentations was chosen to be 100 kbp. This resolution was chosen, since smaller segment sizes would have led to statistical problems resulting from very few counts per segment for many of the elements (genes) looked at, as well as computational runtime limitations. For segment sizes bellow 100 kbp we found that many segments do not contain a single element which could negatively affect our results. Moreover, recent results on violations of Chargaff’s second rule indicate that 100 kbp is an appropriate lower threshold for its validity [29]. While a smaller segment size would, in theory, result in a better resolution of the results, we concluded, based on all these arguments, that 100 kbp is the lowest viable segment size for our investigation. 

In order to avoid stronger biases inside the centromeres caused by lack of genes and other special characteristics, e.g., gaps in the annotated sequence, segments overlapping centromeres were not considered for correlation. Similarly, segments with sequencing gaps (>1% coverage) were removed from the correlation, as they might also produce artifacts by introducing lowered densities of elements and SSTRs not representing the true sequences. 

In some cases, an additional step was taken to mask the impact of SSTRs inside correlated retrotransposons. This was carried out by counting the SSTRs inside of retrotransposons in every 100 kbp segment and subtracting these counts from the empirical SSTR counts within the respective segments, when deriving the densities. Since this method leads to a systematic reduction of SSTR counts, even if there would be no significant enrichment of SSTRs in retrotransposons, we corrected the local counts by adding SSTR counts derived by the length of masked sequences (length of retrotransposons) and the average SSTR count on the respective chromosome.

### 2.3. Nucleotide Models and Significance of Correlations

To test the statistical significance of correlation, we repeated the analysis described above (correlations of local densities with segment sizes of 100 kbp), using repeat contents, predicted by nucleotide-based (first-order Markov) models (as already described in [30]), instead of empirical contents. Using the variance of the modeled results, we derived a significance level, based on the null-hypothesis of tandem repeat contents based on nucleotide frequencies only, by dividing the difference between empirical correlations and model predictions by the respective error approximation (described in [30]).

## 3. Results

### 3.1. G+C Content and Repeat Motif Groupings

**Figure 1:**
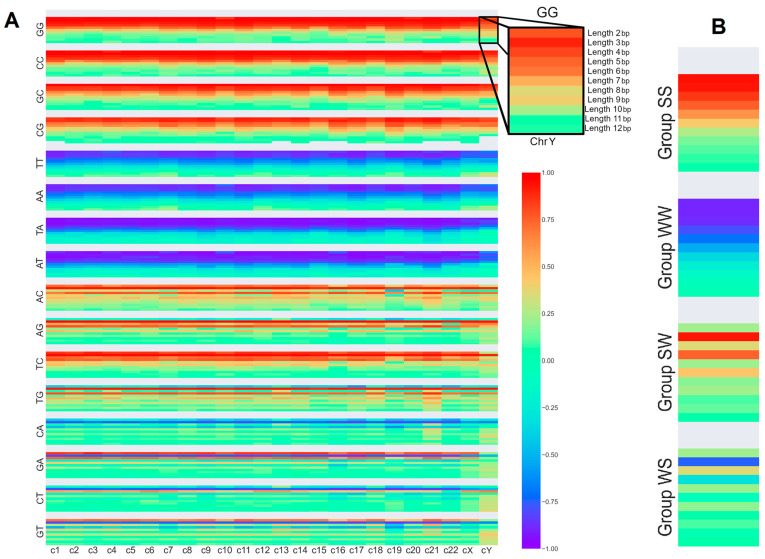
G+C content correlation heatmap. (**A**) shows the full correlation diagram, containing all chromosomes and repeat motifs. The X-axis encodes chromosomal position, and the Y-axis encodes the specific SSTR. The top right has been magnified to clarify SSTR order. (**B**) shows G+C content with grouped motifs and averaged over all chromosomes.

As described above, the G+C content was correlated with SSTRs; Figure 1A shows the full correlation heatmap.

First, the highest variations between chromosomes can be found on chromosomes 19 and Y. Other chromosomes show similar patterns regarding most repeat units and lengths of the SSTRs. For a better overview and easier understanding, we decided for the following correlations with genes and retrotransposons to simplify the data by calculating the averaged correlation of all chromosomes while keeping in mind that single chromosomes may vary from these values.

It is also visible that, while there are strong differences occurring between SSTRs in this correlation, there are also shared patterns between the repeat units. Specifically, there seem to be four groups into which SSTRs may be grouped based on shared patterns visible in Figure 1. These groupings were documented in Table 1. Interestingly, one group is made up of G and C bases only (SS group), and the second group of A and T bases only (WW group). They both show a specific length for which the correlation value shows a maximum (see Figure 1), e.g., in the SS group for length 3 bp on most chromosomes. Increases and decreases in length lead to lower correlation values. Less surprising is that the SS and WW groups consistently result in opposite correlations, with group SS correlating and group WW anticorrelating with G+C content. The third group is made up of mixed WS repeat units, and the fourth group contains the remaining SW units. Both, again, behave similarly, although with a more complex pattern than groups one and two. They oscillate between increases and decreases in correlation with a general tendency towards lower correlations for longer lengths. In Figure 1, this is visible as a distinct striping pattern, which fades away for long lengths. Both groups differ in which lengths correspond to correlation increases and decreases. Considering positive and negative values, group SW increases the correlation for uneven lengths and decreases the correlation value for even lengths. Group WS shows the inverse behavior. It decreases the correlation for uneven lengths and increases it for even lengths. It is also of note that these groups are significantly less homogeneous in their distribution of correlation values compared to the SS and WW groups. While the average values follow the pattern described above, variations may be much higher than in groups SS and WW, and there are a few examples of repeat units not following these patterns (as may be seen in Figure 1).

#### 3.1.1. SSTRs and Genes

**Figure 2:**
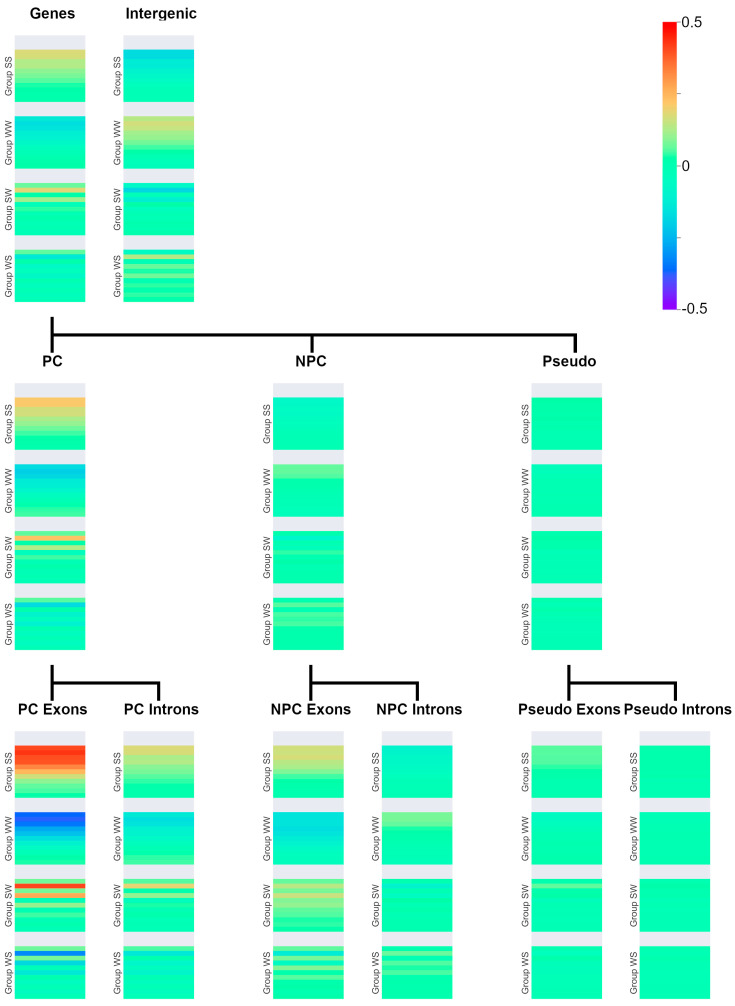
Gene subcategory coverage correlated with SSTRs. The correlation of the four groups of SSTRs with pc, npc, and pseudogenic introns and exons is shown. Significance levels of these results can be found in Figure S3.

While the identified repeat motif groups remained the same regardless of the gene category correlated with the repeat densities, the magnitude and direction of the correlation values changes, depending on the correlation partners. Specifically, there appears to exist three distinct correlation patterns across all examined diagrams (Figure 2).

For example, the first pattern can be seen in the correlation values with genic regions. Visible are patterns similar to the patterns for G+C content correlation. Group SS correlates positively, while group WW correlates negatively. Group SW correlates positively for nearly all odd lengths, while WS correlates negatively for nearly all odd lengths. This pattern will be called G+C-positive, indicating that it is similar to the patterns observed for correlations between SSTRs and G+C content. The second pattern is defined by the inverse behavior, as observed in, for example, intergenic regions. In this case, group SS correlates negatively, while group WW shows a positive correlation. This applies to groups SW and WS as well, which also do switch correlation values. These patterns were called G+C-negative. The last observed pattern is the overall no-correlation case, called G+C-neutral.

Besides the correlation type, correlation strength also varies. Exonic regions of pc genes show the strongest G+C-positive patterns. Exonic regions in pc genes in general seem to correlate in a more G+C-positive manner than their respective counterpart in npc genes. Intronic regions in pc genes do show G+C-positive behavior while intronic regions in npc genes do show, as intergenic regions, G+C-negative behavior. Both exonic and intronic regions in pseudogenes all seem to correlate with very weak G+C-positive behavior or are already showing G+C-neutral behavior.

The length structure is largely consistent across all subgroups, with the maximum intensities of groups SS and WW for lengths 2 and 3 and no special features.

#### 3.1.2. SSTRs and Retrotransposons

**Figure 3:**
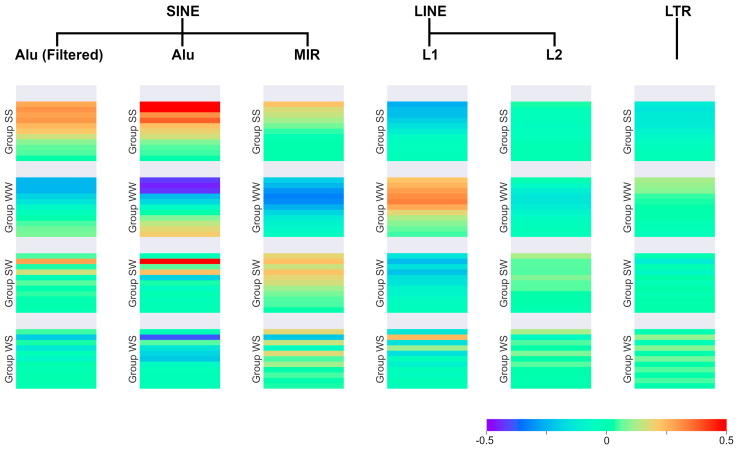
Retrotransposon count correlated with SSTRs. The filtered correlation diagram for SINE Alu is also included. Significance levels of these results can be found in Figure S4.

Lastly, SSTRs were correlated with retrotransposons. For transposons instead of the local coverage, the local counts (number densities) were correlated, as retrotransposons are much shorter and more uniform in length distribution than, for example, introns. Again, the same repeat motif grouping is observed in the correlations. The result is shown in Figure 3.

The separation in G+C-positive and G+C-negative patterns is again applicable, with SINEs, as well as LINE L2, correlating in a G+C-positive manner and LINE L1 correlating in a G+C-negative manner. Lastly, LTR correlates mostly in a G+C-neutral way.

However, this time, there are some significant deviations from the expected observations. LTRs show a very weak correlation in the averaged diagram. However, looking at the full correlation heatmap shows strong G+C-negative behavior mostly on chromosome 19, and some others on chromosome 16, 17, and 20, as well as on 21 (see Supplementary Figure S1), while the rest of the genome correlates only weakly or not at all. There are also anomalies in the length structure, specifically the group WW behavior of SINE Alu. The expected behavior would be a maximum correlation intensity for length three, and then a steady decrease in intensity for longer lengths towards no correlation for longer lengths.

Instead, after reaching no correlation at around length 7 bp, the correlation type flips, and intensity starts increasing again. The full correlation heatmap shows that this effect only occurs for (A)_n_ and (T)_n_, while (AT)_n_ and (TA)_n_ do not show this pattern. One possible explanation for this is the structure of Alu. It is made up of two monomers connected by a region rich in A [5]. It would, therefore, be plausible to assume that this is the cause of an overabundance of mono-A stretches (or mono-T stretches on the opposing strand) near Alu sequences, and, therefore, this would reveal a correlation between long (A)**_n_**, (T)**_n_** and Alu.

To verify this assumption, the impact of the SSTRs inside of the Alu segments on the correlation values was minimized. This was carried out by masking the density of SSTRs inside Alu. This was carried out by searching for the SSTRs in Alu and replacing their density by the average SSTR density on the chromosome. Therefore, the impact of these areas on the correlation value is removed. The leftmost column in Figure 3 shows the filtered correlation for Alu.

As predicted, the anomalous behavior of Alu stops, returning to expected values. Applying the same procedure to all other retrotransposons resulted in no significant changes, aside from an overall decrease in correlation strength (see Supplementary Figure S2).

## 4. Discussion

### 4.1. Chromosomal Properties

The correlations of SSTRs with G+C content has been shown on the chromosomal level in Figure 1. Variations are found, especially on chromosomes for which specific properties are well-known, such as, for example, chromosome 19 as the richest in genes or chromosome 21 as the shortest autosome, as well as chromosome Y as the gonosome with very specific properties regarding structure and gene density. Accordingly, these properties may also be considered for an explanation of these variations. It might be interesting for further research to check for the correlation behavior on shorter sequence parts, as the arms of a chromosome or the centromeric region. While we do not expect large differences, using telomere-to-telomere sequences for further analysis could also be interesting.

### 4.2. Repeat Motif Groups

One key finding of this article is the identification of four specific repeat motif groups, which share similar correlation patterns with all genic elements and retrotransposons. In the following, we will discuss to what extent certain observed patterns can be explained as the results of known sequence constraints, and we identify independent (yet unknown) patterns.

When looking at the entire human genome, it is expected that very short DNA sequences and their reverse complementary counterparts should always appear at roughly equal rates. In the case of the length k = 1, this is stated in Chargaff’s second rule [31]. For *Homo sapiens*, this reverse complementary symmetry has been, at least, shown to hold up reasonably well for *k-mers* of lengths of up to k = 7 [32]. This rule can be used to explain many characteristics shared between members within groups SS and WW. Reverse complementary SSTRs (e.g., GCGC and CGCG) always belong to the same group. Therefore, SSTRs with reverse complementary repeat units (e.g., AG and CT) are expected to always show the same patterns for all values of k.

While this might explain many of the observed patterns, two observed features are not explained by this. While similar patterns between GG and CC, as well as similar patterns between CG and GC, were expected, the similarities between CC/GG and CG/GC were not a direct consequence of the similar contents of reverse complement sequences. Second, reverse complements do not explain the inverse correlation type between group SS and WW observed for all correlated sequences. One possibly helpful observation is that, inside group SS and WW, respectively, SSTRs share the same extreme G+C contents (100% in group SS, and 0% in group WW). Therefore, the differences in G+C content might be the source of the observed correlations, treating all SSTRs inside either group SS or WW equally. This line of thinking would also explain the opposite correlation between groups, as the G+C content flips between groups.

If the G+C content would be the only determining factor, SSTRs within groups SS and WW would show similar patterns, for all lengths, as their G+C content is always the same. Figure 1B correlates with the G+C content and shows a complex length structure distribution, implying that the connection is more complex and the differences in G+C content cannot alone explain our observations.

Groups SW and WS can be discussed analogously. However, the relationships between the repeat units are more complex. First, reverse complementary repeat motifs are no longer in the same motif group (e.g., GT is in SW and AC in WS). Second, reverse complementary SSTRs were considered as SSTRs with the reverse complementary repeat units, for even lengths (e.g., for TCTC and GAGA, the repeat units are TC and GA, respectively). For odd lengths, the reverse complementary SSTRs were considered as SSTRs with the complementary (but not reversed) repeat unit (e.g., for TCTCT and AGAGA, the repeat units are TC and AG, respectively). Accordingly, for even lengths, the reverse complementary SSTRs do not belong to the same group, while they do belong to the same group for odd lengths.

Taking this into account and under the assumption of the extension of Chargaff’s second rule as mentioned above, we would expect correlation patterns different from the observations for SS and WW groups. SSTRs of odd length would be indistinguishable from reverse complementary SSTRs at the same genomic position but on differing strands. For SSTRs of even length, this is not the case, and, therefore a weaker correlation, if any, would be expected. Interestingly, violations of Chargaff’s second rule for oligonucleotides were also found in another recent study [29].

Considering correlations of SSTRs and G+C content, for groups SS and WW, the G+C content is the same for all SSTR lengths (either 0% or 100%). Group SW and WS members, however, have a mixed repeat unit, meaning they contain one G/C and one A/T base. Therefore, the G+C content of their SSTRs oscillates depending on length. SSTRs of even length will always have a balanced G+C content of 50%. However, for SSTRs of odd length, the G+C content varies. It starts with the most slanted values, with length 3 being roughly 66.6% or 33.3%, depending on the repeat unit group. The amplitude of this oscillation decreases for longer odd lengths. Regarding G+C content, this means that SSTRs of even length with a balanced G+C content do not correlate or anti-correlate significantly, while the high correlation values with the slanted G+C content produce higher correlation or anti-correlation intensity stripes. This pattern is observable in Figure 1. This could also explain the fading effect of the stripe pattern for longer lengths, as the underlying SSTRs are more and more balanced in terms of G+C content as they increase in length. This may be seen in addition to a general decrease of correlation for longer SSTR lengths visible for groups of SS and WW.

The G+C content might also explain why the correlation types of groups SW and WS seem to be related to the correlation types of groups SS and WW, respectively. Odd lengths of group SW correlate in the same direction as group SS (positively) and both have G+C contents above 50%. Odd lengths of group WS correlate in the same direction as group WW (negatively) and some have lower G+C contents. With the G+C content as the dominating factor for the observed patterns, one would expect that both groups correlate in the same direction. Similar things hold true for groups WS and WW. If the observed patterns would depend on G+C content only, then there should be no meaningful difference between repeat units with an identical G+C content, e.g., between (GA)_n_ and (CA)_n_. In fact, groups SW and WS show a large variance between group members and also between members of different groups with an identical G+C content; e.g., correlations between TC and G+C content are length-independent for short lengths, while correlation values for GA also oscillate strongly for short lengths (See Figure 1A). The findings, especially in group WS, strongly illustrate specific length-dependent correlation values as, for example, GA as well as GAGA correlate strongly positively but GAG strongly negatively. This is far stronger than what has been seen as, for example, in group SW, and is especially striking as GAG is higher in G+C content in comparison with GA. This illustrates impressively the length dependencies of SSTRs and proves patterns independent of pure G+C content as an explanation. Summing up, it was shown that SSTR motifs can be grouped into four groups based on their patterns in correlation with G+C content. The highest correlation and anti-correlation values are mostly found for the shortest SSTRs. A significant part of the similarities can be explained by assuming reverse complement symmetry. However, not all structures are explained by this. While it is likely that G+C content is an important contributor, we found features that cannot be explained by either the G+C content or symmetry between strands, such as the length structure or variance inside groups.

### 4.3. Genic Categories

There are multiple things of note about the observed correlation pattern. First, it is nontrivial that nearly all genic regions seem to show correlation patterns being of either G+C-positive or -negative behavior, with only one exception for pseudogenes and their subcategories presenting no correlation with SSTRs. Additionally, there seem to be differences in correlation type based on genic properties. First, pc genes are correlating strongly with G+C positively and npc genes slightly negatively, and, second, with exons and introns also correlating differently. Understanding where exactly these patterns originate from and what they imply is beyond the scope of this article and needs further research. However, some observations can be made. Exons show G+C-positive patterns for both pc and npc genes, while introns show G+C-positive patterns for pc genes and G+C-negative for npc genes. This is interesting as it would contradict interpretations such as transcribed areas being G+C-positive, with non-transcribed areas being G+C-negative. On the other hand, if exonic and intronic correlation is looked at relatively to their higher-order category (e.g., pc genes for pc exons; see Figure 2), then, in both cases, exons show more G+C-positive patterns than their parent gene, and their introns show more G+C-negative patterns. Overall, genes correlate in a G+C-positive manner while intergenic areas are G+C-negative and allow for the hypothesis that transcribed areas are more G+C-positive than their environment, while the reverse is true for non-transcribed areas. In most cases, the maximum correlation strength is somewhere between lengths 2 bp and 4 bp in genic regions, but some difference may be observed, as, for example, for pc exons and pc introns. The stripe pattern for different lengths in groups SW and WS is again visible. As discussed above, G+C content is likely relevant for some of the SSTR motif groupings. It is, therefore, plausible that the (well-known) generally higher G+C content of genes is one main determinant of this pattern, while it cannot explain certain details. While we see a formal analysis of the relation as outside of the scope of this article, considering the observed deviations from Chargaff’s second rule, it seems plausible that the SSTR densities are a driving force of recently discovered deviations of Chargaff’s second rule in coding sequences [29] or that this violation affects SSTR densities.

### 4.4. Retrotransposons

As observed for genic categories, retrotransposons also seem to be connected to SSTR distributions, as all retrotransposons show some amount of correlation. However, it is not easy to explain why certain retrotransposons behave G+C-positive or negative. Both SINEs, Alu and MIR, correlate G+C-positive. The LINE L1 correlates G+C-negative and L2 correlates nearly G+C-neutral with slight positive tendencies, showing that retrotransposons of the same group can correlate differently.

One possible explanation would be the proximity of retrotransposons to genic regions. L1, for example, negatively correlates with genes [5] while also showing G+C-negative behavior, while Alu tends to be in the proximity of genes [5] and is also behaving G+C positively. Applying this logic, the correlation of retrotransposons and SSTRs is only the results of an indirect connection with SSTRs.

One notable counterargument against this theory is the observed difference in length structure visible between retrotransposons and genic categories for some repeat motif groups. If retrotransposons correlate because of proximity to genes, their length structure should be the same when correlating SSTRs with G+C content or genes directly. In fact, we observed differences for e.g., for L1 in the WS or WW group.

Summing this all up, SSTRs show complex, length dependent correlation patterns with both genic regions as well as retrotransposons. The causality of this connection remains unknown. However, some tendencies, such as increasingly G+C-positive correlation for transcribed areas, suggest a link between transcription and SSTR density.

### 4.5. SINE Alu

The final observation to discuss is the anomalous patterns of SINE Alu. As discussed above, nearly all correlated sequences show either G+C-positive or -negative patterns, varying only in the correlation strength and minor differences in the length structure. However, SINE Alu breaks this pattern for its correlation with group WW. Instead of an expected decreasing, negative correlation for longer SSTR lengths, Alu correlation increases towards positive correlation values. Masking the sequences inside of Alu elements removes these anomalies, thus leading to the conclusion that the sequence of SINE Alu is the source of these patterns (Alu sequences contain poly-A stretches [5]), while the environment around Alu shows the expected patterns. As this pattern has not been found for other retrotransposons, it points towards a specific property of Alu. Alu is not only an element correlating with SSTRs, but it carries SSTRs and partially dominates its SSTR environment.

## 5. Conclusions

The results clearly indicate a complex non-random structure in the positional distribution of SSTRs. The relevance of SSTRs is further supported by the correlation of functional features such as the G+C content, genes, and retrotransposons with densities of specific SSTRs in their environment. Considering SSTRs, especially of lengths of up to 4 or 5 bp, as functionally interacting with elements as genes or retrotransposons in their environment would open a new avenue for considerations on gene and genome regulation.

For genic areas, both pc and npc genes show a connection to their SSTR environment, while pseudogenes do not. This would allow for the conclusion that SSTRs are connected to whether an area is transcribed or not, thereby implying a connection between SSTR environment and gene expression. This is supported by exons correlating more intensely than genes overall, again showcasing that a stronger connection to transcribed areas correlates with a more strongly modified SSTR environment.

An interesting consequence of this would be that, independently of the specific process that turns a gene into a pseudogene, they do not only lose their functionality, but associated changes in their sequences also alter their influences on the local SSTR environment. While our observations cannot identify a causal relationship between SSTRs and pseudogenization, the analysis of SSTR environments around pseudogenes that lost their functionality recently (on evolutionary time scales) and still-functional genes could give interesting insights. While such an analysis is out of the scope of this study, our observations motivate further research on that topic.

For retrotransposons, there seems to be a connection between LINEs, SINEs, and SSTRs, while LTRs seem mostly unrelated. This further supports a connection between SSTR distribution and a specific functionality, as retrotransposons were also known to regulate gene expression. The connection seems to be complex in nature, as related sequences as, for example, L1 and L2 from the LINE family correlate in opposite ways. At the same time, certain retrotransposons correlate with genes themselves, making it difficult to decide whether the observed patterns are connected to the gene and SSTR connection mentioned above or are an independent phenomenon based on SSTR and retrotransposon interaction. One especially interesting case in this line of arguments is that of Alu in the SINE family. The results have shown that the Alu sequence itself has a strongly anomalous SSTR makeup, thereby significantly impacting the SSTR environment. In that sense, Alu could be seen as a carrier for a specific SSTR environment. If there is a connection between SSTRs and functionality, then “SSTR carrier” sequences like Alu could move these environments to a specific place inside the genome, where the provided effect is needed. If this was the case, it should be possible to find similar patterns inside genomes of other species. This could be checked, by conducting the same analysis performed in this article on other genomes. Another interesting avenue would be the analysis of other elements, which could act as SSTR carriers like Alu. An especially interesting variation of this would be to look at species that do not possess Alu but seemingly equivalent elements and search for sequences that transport a similar SSTR profile.

Besides the relationships between SSTR groups and other elements, the existence of SSTR groups and the relation between patterns of different groups is nontrivial. As mentioned above, some of these patterns can be explained by the symmetry of reverse complementary *k-mers* and a possible connection to G+C content. However, many patterns cannot be explained that easily and require further research. Parts of the observed length structures, especially, as well as chromosomal differences, are not linked to these explanations. Chromosome 19, for example, showed an anomalously strong correlation, while, again, a link to gene functionality might be the reason for this anomaly, as chromosome 19 is known to be very gene-rich.

From all these findings it is obvious that many possible relations and interactions of SSTRs with chromosomal and genic regions may be worth further researching in order to better understand the properties and possible functionalities of SSTRs and how they contribute to genic or genomic regulation.

## Figures and Tables

**Table 1:** Overview of SSTR groups based on the patterns of the Pearson correlation coefficient in the correlation with G+C content.

(SS)_n_	(WW)_n_	(SW)_n_	(WS)_n_
(CC)_n_	(AA)_n_	(GA)_n_	(AG)_n_
(GG)_n_	(TT)_n_	(CA)_n_	(AC)_n_
(GC)_n_	(AT)_n_	(GT)_n_	(TG)_n_
(CG)_n_	(TA)_n_	(CT)_n_	(TC)_n_

## Data Availability

All codes and scripts (including visualization) used for this article, as well as a manual, are available online at http://www.kip.uni-heidelberg.de/biophysik/software or from an associated GitHub repository (https://github.com/Sievers-A/Oligo, accessed on 27 September 2021).

## Supplementary Materials

The following supporting information can be downloaded at: https://www.mdpi.com/article/10.3390/genes15010033/s1, Table S1: List of RefSEQ IDs, Figure S1: Full correlation diagram for LTRs, Figure S2: Filtered and unfiltered correlation of retrotransposons, averaged over SSTR motif groups and all chromosomes, Figure S3: Significance levels of gene subcategory coverage correlated with SSTRs, Figure S4: Significance levels of retrotransposon count correlated with SSTRs.
