# Supplementary material for: Specific Patterns in Correlations of Super-Short Tandem Repeats (SSTRs) with G+C Content, Genic and Intergenic Regions, and Retrotransposons on All Human Chromosomes
Source: Genes (Basel). 2023 Dec 25;15(1):33. doi: 10.3390/genes15010033 (PMC10815669; doi:10.3390/genes15010033)
Supplement: Supplementary file 1 [file genes-15-00033-s001.zip › genes-2770107-supplementary.pdf]

Supplementary Data

Table S1: List of RefSeq IDs for all used genbank files of the homo sapiens genome GRCh38.p13.

Chromosome	RefSeq ID
1	NC_000001.11
2	NC_000002.12
3	NC_000003.12
4	NC_000004.12
5	NC_000005.10
6	NC_000006.12
7	NC_000007.14
8	NC_000008.11
9	NC_000009.12
10	NC_000010.11
11	NC_000011.10
12	NC_000012.12
13	NC_000013.11
14	NC_000014.9
15	NC_000015.10
16	NC_000016.10
17	NC_000017.11
18	NC_000018.10
19	NC_000019.10
20	NC_000020.11
21	NC_000021.9
22	NC_000022.11
X	NC_000023.11
Y	NC_000024.10

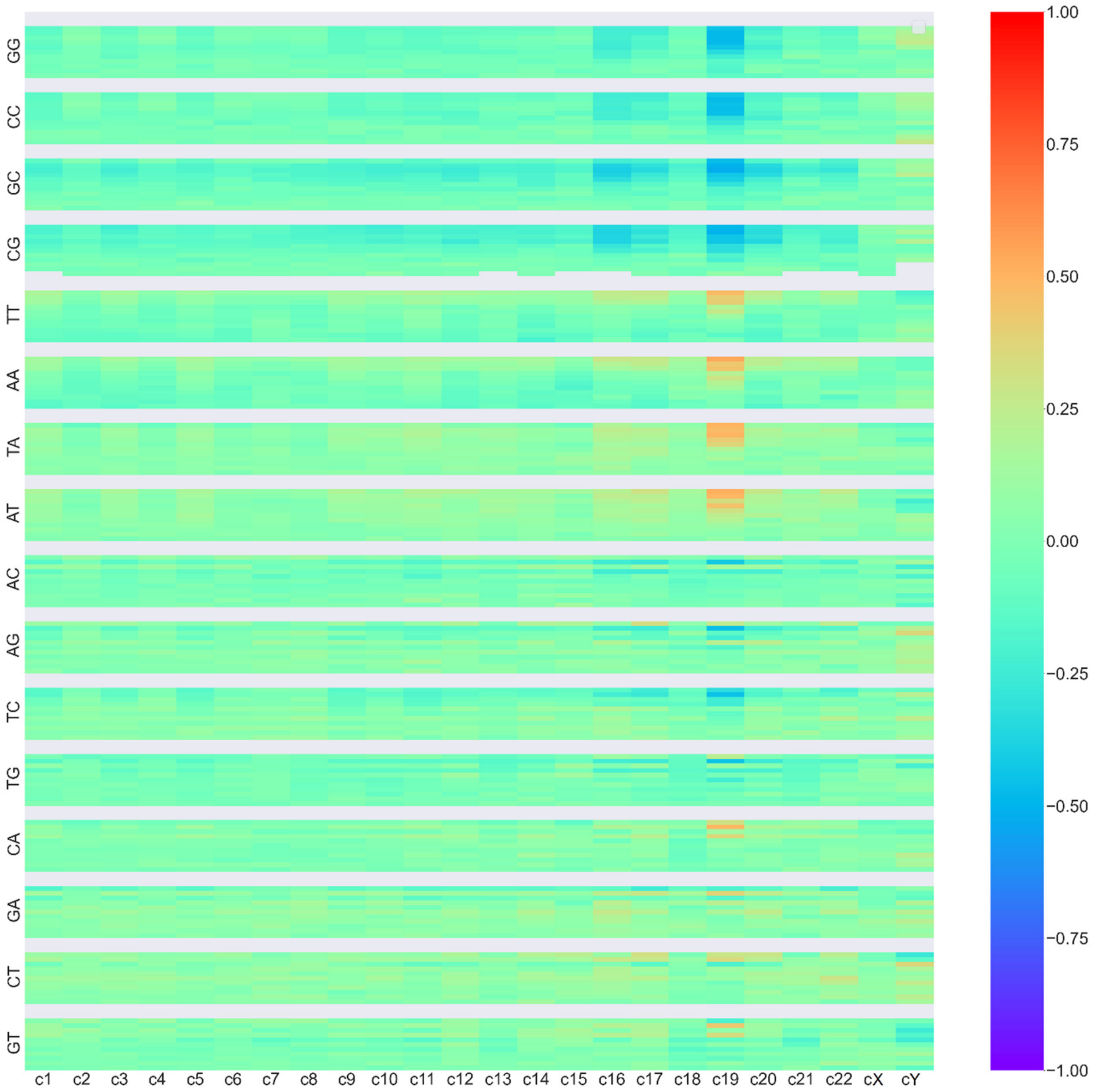

Figure S1. Full correlation diagram for LTRs. Chromosome 19 shows anomalous behavior, breaking the observed pattern of all chromosomes behaving mostly the same for most correlation pairs.

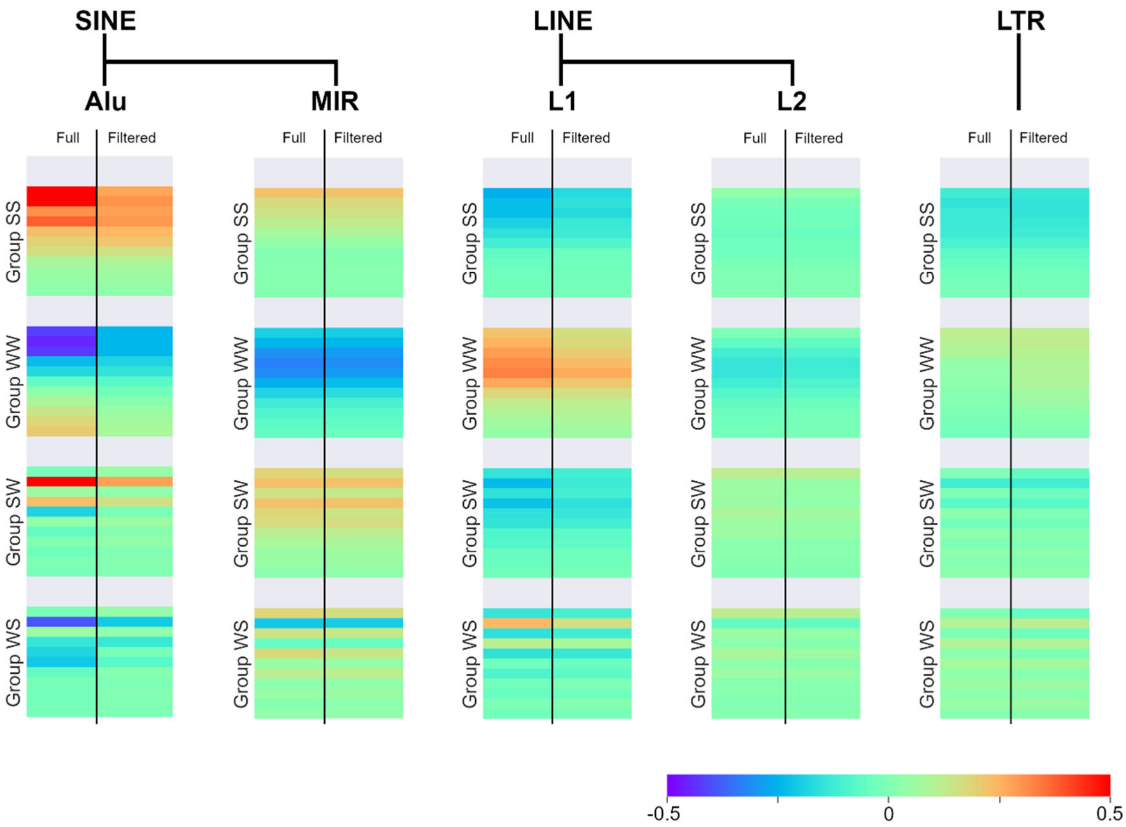

Figure S2. Filtered and unfiltered correlation of retrotransposons, averaged over SSTR motif groups and all chromosomes. The left side of each correlation shows the unfiltered results. The right side shows the results if all SSTRs inside the correlated retrotransposon are ignored and replaced by the average chromosomal retrotransposon density of the given type. In general, a small decrease in average correlation intensity can be observed. Special attention was paid to the WW Group for Alu, as it changes its behavior for longer chains. Unfiltered results show positive correlation for longer chains, while filtered results show the expected behavior of no correlation for longer lengths.

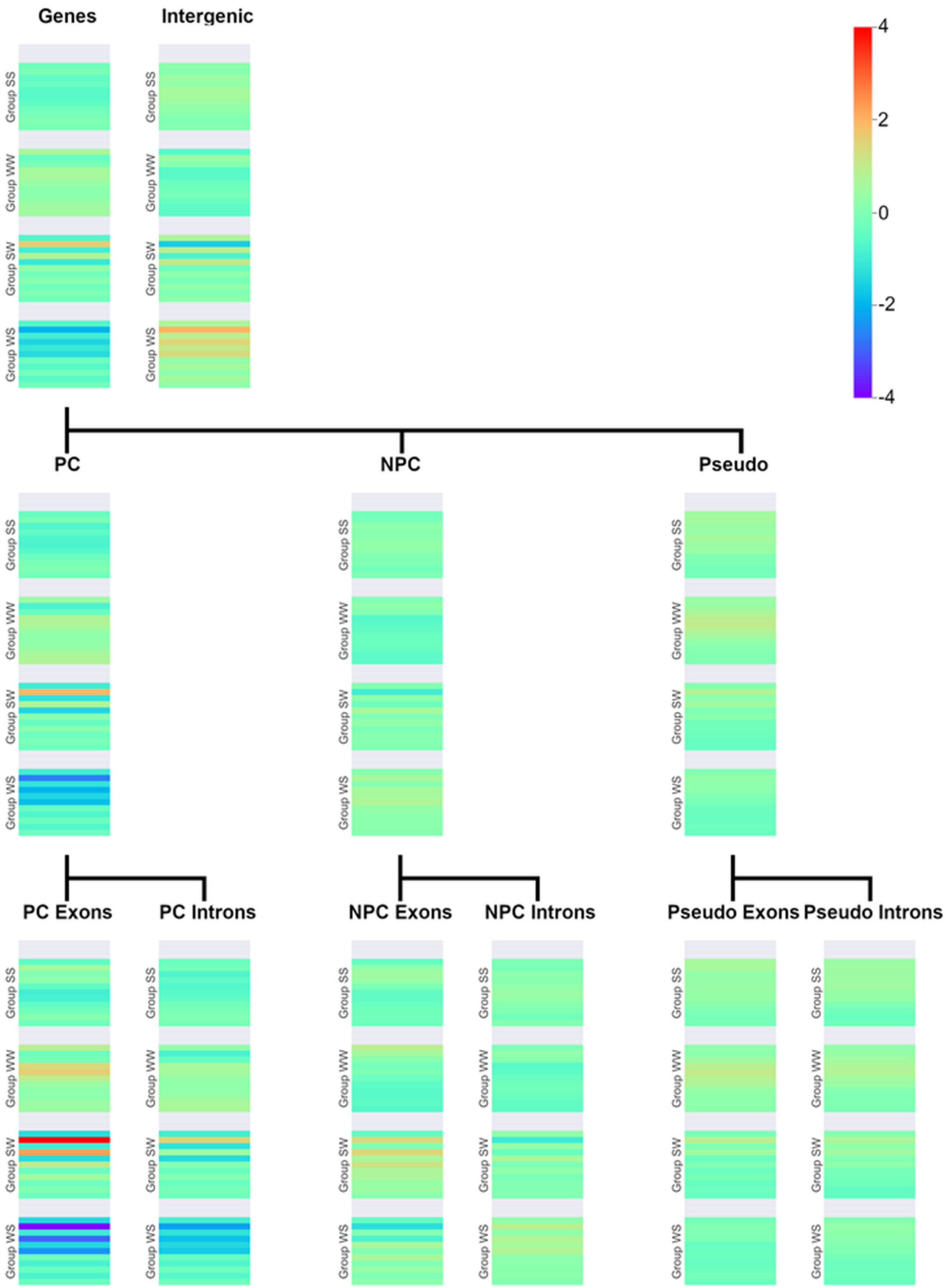

Figure S3. Significance levels of gene subcategory coverage correlated with SSTRs. The correlation of the four groups of SSTRs with pc, npc and pseudogenic introns and exons is shown.

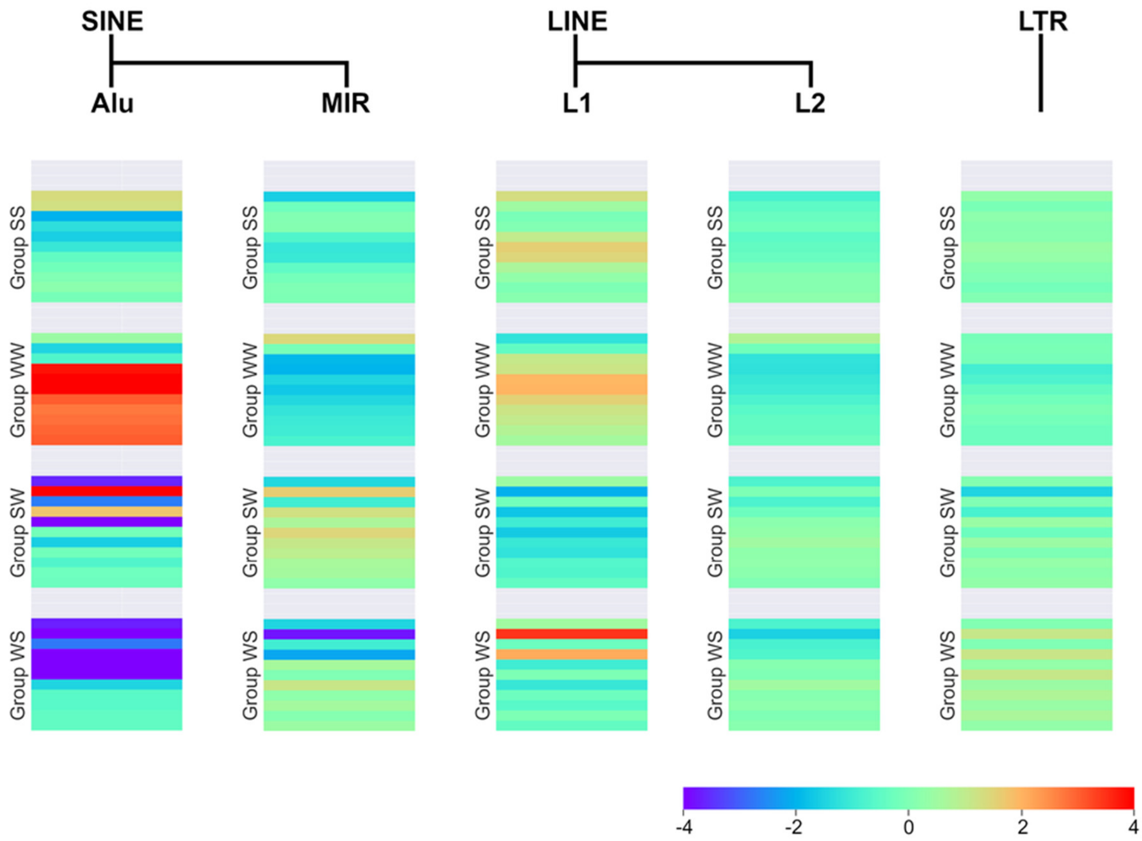

Figure S4. Significance levels of retrotransposon count correlated with SSTRs. The filtered correlation diagram for SINE Alu is also included.
